# Acceptability and Feasibility of Health Measures in Preteens: Findings From the ROLO Longitudinal Birth Cohort Study

**DOI:** 10.1111/hex.70359

**Published:** 2025-07-28

**Authors:** Sophie Callanan, Anna Delahunt, Rachel K. Crowley, Patrick J. Twomey, Catherine M. Phillips, Alex O. Start, Ciara M. McDonnell, Declan Cody, Fionnuala M. McAuliffe

**Affiliations:** ^1^ UCD Perinatal Research Centre, School of Medicine University College Dublin, National Maternity Hospital Dublin Ireland; ^2^ Department of Endocrinology St Vincent's University Hospital Dublin Ireland; ^3^ Department of Clinical Chemistry St Vincent's University Hospital Dublin Ireland; ^4^ School of Public Health, Physiotherapy and Sports Science University College Dublin Dublin Ireland; ^5^ Department of Paediatric Endocrinology & Diabetes Children's Health Ireland, Temple Street Hospital Dublin Ireland; ^6^ Department of Diabetes & Endocrinology Children's Health Ireland, Crumlin Dublin Ireland

**Keywords:** feasibility, health research, research methods, young people

## Abstract

**Introduction:**

Limited research has investigated young people's opinions on health measures that are used in clinical and research settings. This study aimed to describe young people's views on research methods utilised in a longitudinal birth cohort. It also aimed to explore the feasibility of (i) blood pressure and a fitness assessment as a substitute for blood biomarkers; (ii) foot length as a substitute for maternal‐reported stage of puberty and (iii) neck and mid‐upper arm circumference as a substitute for body composition analysis in preteens (9–11‐year‐olds).

**Methods:**

This is a mixed‐methods analysis of preteens (*n* = 408) who were born into the ROLO longitudinal birth cohort study. Weight, height, skinfold thickness, circumferences, body composition, blood pressure, fitness (shuttle run test score), blood biomarkers, stage of puberty and foot length were obtained at 9–11 years of age. A subgroup completed a Likert‐style acceptability questionnaire after their study visit and took part in public and patient involvement to gain further insight into opinions of health measures utilised. Statistical comparisons and linear regression models explored associations in the total group and stratified by child sex. Results were adjusted for multiple testing.

**Results:**

Of 50 preteens who completed an acceptability questionnaire after their study visit, no participants rated the study measurements, exercises or questionnaires as unacceptable research methods, and only 5% rated providing a blood sample as unacceptable. Blood pressure percentiles and fitness scores were not strongly associated with blood biomarkers. In the total group, after adjustment for multiple testing, neck and mid‐upper arm circumference were positively associated with body mass index *z*‐score (Beta [*B*] = 0.42, 95% confidence interval [CI] = 0.34,0.49, *p* < 0.001, *q* = 0.015; *B* = 0.32, 95% CI = 0.29, 0.35, *p* < 0.001, *q* = 0.005), waist circumference (cm) (*B* = 2.83, 95% CI = 2.22, 3.45, *p* < 0.001, *q* = 0.016; *B* = 2.10, 95% CI = 1.78, 2.41, *p* < 0.001, *q* = 0.010) and body fat (%) (*B* = 2.26, 95% CI = 1.66, 2.87, *p* < 0.001, *q* = 0.018; *B* = 2.05, 95% CI = 1.80, 2.30, *p* < 0.001, *q* = 0.008), respectively.

**Conclusion:**

Research methods were acceptable to preteens. Neck and mid‐upper arm circumference may serve as simple and less burdensome indicators of adiposity. Future research should explore minimally invasive blood sampling techniques.

**Patient or Public Contribution:**

Preteens contributed to the manuscript analysis by sharing their insights and ideas related to health measurements used in a longitudinal birth cohort study. Their input provided important perspectives on the acceptability of health measures in this age group that may have important implications for researchers and clinicians conducting health research with youth.

**Abbreviations**
B: Beta
BMI: body mass index
CI: Confidence interval
DXA: Dual‐energy X‐ray absorptiometry
HDL‐C: High‐density lipoprotein cholesterol
NMH: National Maternity Hospital
ROLO: Randomised cOntrol trial of a LOw glycaemic index diet in pregnancy to prevent macrosomia
SD: standard deviation
YPAG: Young Person's Advisory Group

## 1 Introduction

Young people who participate in health‐related research are pivotal for the improvement and evolution of medical care [1]. Understanding the experience of medical research subjects is critical to support current and future research activities, and participants want their voice to be heard [2, 3]. Their feedback can enhance the patient‐centredness of future trial design and delivery [4], support long‐term retention by increasing the likelihood of future research participation [5], and strengthen the validity and rigour of future research outputs [6]. These factors combined may have direct implications for patient satisfaction and quality of care in clinical settings. Limited qualitative literature that documents the views of youth suggests that minimally invasive health assessment techniques with low burden are preferred [1, 2], although more evidence is needed.

Blood sampling is one of the biggest barriers to youth participation in medical research and is often considered frightening, unpleasant or one of the worst parts of study participation [1]. Needle phobia can cause significant distress and lead to long‐term avoidance of healthcare [7]. Recent research has reported that the pain associated with venous puncture is stressful and burdensome for children and their parents, compared to other procedures used in clinical trials [8]. Previous studies have identified blood pressure and cardiorespiratory endurance testing as enjoyable research methods for youth, associated with low aversion [1, 9]. Both parameters are inexpensive, painless and are associated with the cardiovascular and metabolic profiles of children and adolescents [10, 11].

Procedures related to sexual development have also been described as particularly uncomfortable and burdensome for youth taking part in research [1]. Quick assessments that require minimal physical touch by the professional and that do not require complete removal of clothes are preferred [12]. Tanner stage assessment has been regarded as embarrassing by youth and offensive by some schools and parents and is also subject to inaccuracies from self‐report and privacy concerns [13, 14]. Foot size may serve as an easier and less invasive predictor of sexual maturity that could be incorporated into health assessments, which is considered acceptable to youth [12, 15, 16, 17].

Thus far, there has been limited qualitative research investigating young people's opinions on measures of obesity in health research [18], which is important given possible body image concerns in this age group [19]. A previous elicitation study with 12‐year‐olds reported that a large proportion experienced embarrassment or adverse reactions to being weighed or having their waist circumference measured [18]. While dual‐energy X‐ray absorptiometry (DXA) is the gold standard of body composition assessment, that is well tolerated due to its open configuration and short testing duration, the large‐scale availability of such complex equipment is often not feasible [20]. Alternative methods, such as circumferences of the neck and arm, have been shown to discriminate body fat and may serve as suitable and practical tools for preteens in healthcare and research settings [21, 22].

Acceptability and ease of use of research methods are important factors when considering their feasibility as standard methods of health assessment. Most research to date has captured the views of adult participants or the parents of children involved in health research [23, 24], and there is a stark paucity of literature collecting the perspectives of young people themselves. Many qualitative studies focus on the motivations and barriers influencing participation in clinical trials or only include those living with chronic health conditions [25]. There is a lack of research gathering opinions on the methods and tools being utilised in research, particularly in longitudinal birth cohorts, which is important given these studies rely heavily on long‐term retention and the same techniques are often used at each follow‐up study [5, 6, 26]. Furthermore, previous studies have collected qualitative or quantitative data, and few have combined methods to maximise insights into the participant experience [8].

This mixed‐methods research aimed to fill this gap by qualitatively describing young people's views on research methods utilised in a longitudinal birth cohort study. It also aimed to assess how standard measures of health for preteens correlate with less invasive options that could be used in healthcare and research settings to reduce burden. Therefore, we quantitatively explored the feasibility of (i) blood pressure and cardiorespiratory endurance as a substitute for blood biomarkers; (ii) measurement of growth parameters as a substitute for maternal‐reported sexual maturity; and (iii) measurement of neck and mid‐upper arm circumference as a substitute for body composition analysis using data collected from a cohort of Irish preteens born into the ROLO (Randomised cOntrol trial of a LOw glycaemic index diet in pregnancy to prevent macrosomia) longitudinal birth cohort study. We hypothesise that research methods will be acceptable to preteens and that less invasive measures of health will correlate with standard methods of health assessment.

## 2 Methods

### 2.1 Subject Design and Selection

This is a mixed‐methods analysis of preteens born into the ROLO study [27]. In brief, eligible participants of the primary study were healthy women attending the National Maternity Hospital (NMH), Dublin, Ireland (2007–2011), for their second pregnancy who previously delivered an infant with macrosomia (birthweight > 4 kg). The NMH is a large tertiary care setting with a total bed capacity of 164, which was chosen as the single recruitment site because it provided access to a diverse and large volume of patients. The prevalence of neonates born with macrosomia is also higher in the NMH, compared to other tertiary care settings in Dublin [28].

At the onset of the original study, a power calculation was performed to establish the number of participants that were required to show a 0.25 standard deviation (SD) (equivalent of 102 g) difference in birthweight between the intervention and control groups. The study was powered for 360 participants in each group at a significance level of 5% and a power of 90%. A total of 800 women were recruited to the study to account for early dropouts or potential loss to follow‐up during pregnancy. A simple randomisation approach using allocation concealment was used, where a research midwife met and randomised each woman into the intervention or control arm of the trial using computer‐generated allocations in a ratio of 1:1. Women were not eligible to participate if they were under 18 years old, had a multiple pregnancy, or had a history of gestational diabetes mellitus in their current or previous pregnancies. Those with underlying medical conditions that required medication or that had in utero growth abnormalities detected during their second pregnancy were also excluded.

The intervention component included a low glycaemic index dietary education session provided by a research dietitian in groups of 4–6 participants within 2 weeks of randomisation (mean [SD] gestation 15.7 [3.0] weeks). The research dietitian met with intervention subjects again at 28 and 34 weeks' gestation for brief reinforcement. The control group received routine antenatal care, which did not include formal dietary advice. Both groups completed 3‐day food diaries and health and lifestyle questionnaires at three timepoints in pregnancy. At delivery, data was obtained for 759 dyads. Mother–child pairs from both arms of the trial have been followed up throughout childhood as part of the ROLO longitudinal birth cohort [29]. When each ROLO child turned 3 months; 6 months; and 2, 5 and 9–11 years of age, the research team contacted mothers to invite them and their child to participate in a follow‐up visit.

### 2.2 Eligibility to be Included in the Present Analysis

Participants were eligible to be included in the present analysis if they were born into the original ROLO study and attended a follow‐up visit at 9–11 years of age (ROLO Preteen study), regardless of whether they had participated in previous follow‐up studies. Of 437 dyads who participated in the ROLO Preteen study either remotely (completed questionnaires only) or in‐person, those who obtained at least one measurement at the in‐person study visit were analysed, which resulted in a total sample size of 408 preteens. Participants were excluded if they did not attend an in‐person visit.

### 2.3 Informed Consent and Ethics

Informed, written maternal consent was obtained before study participation, and verbal assent was obtained from the study child. Ethical approval for the primary ROLO trial was obtained from the Ethics Committee of the National Maternity Hospital in December 2006. Ethical approval for the 9–11‐year follow‐up was obtained from the University College Dublin Office of Research Ethics Committee in February 2015. Data collected from this birth cohort study is permitted to be stored and managed indefinitely with consent.

### 2.4 Acceptability of Research Methods in the ROLO Preteen Study

#### 2.4.1 Acceptability Questionnaire

A subgroup of preteens (*n* = 50) completed a self‐reported acceptability questionnaire post study visit that consisted of four questions on a 5‐point Likert scale from 1 (‘completely unacceptable’) to 5 (‘very acceptable’). It explored whether the preteen found the ROLO study measurements, exercises (20‐m shuttle run test), online health and lifestyle questionnaires, and blood sample acceptable.

#### 2.4.2 Public and Patient Involvement

The ROLO Young Person's Advisory Group (YPAG) consists of ROLO study children and their older siblings who meet at least annually with the research team. A detailed description of the methodology and previous findings has been published previously [9]. In brief, the fifth YPAG meeting was held in University College Dublin in April 2023 for the first time in person since the group was established in 2020. The researchers prompted the group to share their opinions on research methods that were used in the ROLO Preteen study. There was insufficient content to conduct formal thematic analysis; therefore, the researchers summarised the views shared during this session.

### 2.5 Health Measurements at 9–11 Years of Age

#### 2.5.1 Anthropometry and Body Composition

Weight was measured to the nearest 0.1 kg using a calibrated weighing scale, and height was measured to the nearest 0.1 cm using a portable stadiometer (SECA GmbH & Co. KG. Hamburg, Germany). Body mass index (BMI) was calculated, and values were converted to *z*‐scores according to the 1990 UK reference data [30, 31, 32]. Circumferences of the mid‐upper arm, neck, hip and waist (at the point of the umbilicus) were measured to the nearest 0.1 cm using an ergonomic circumference measuring tape (SECA GmbH & Co. KG. Hamburg, Germany). The sum of skinfolds was determined from three sites (biceps plus triceps plus subscapular) to the nearest 0.2 mm using the Holtain Tanner/Whitehouse callipers (Holtain Ltd, Crymych, the United Kingdom). DXA was performed using the Lunar iDXA scanner (GE Healthcare, Madison, Wisconsin, the United States) with enCORE v.18.0 software to obtain total fat mass (kg) and percentage body fat. Visceral adipose tissue was estimated using sex‐specific prediction equations (*R* ^2^: girls = 50.8%; boys = 55.8%; standard error of the estimate: girls = 13.5 cm^2^; boys = 13.4 cm^2^) [33].

#### 2.5.2 Blood Pressure Measurement

Systolic and diastolic blood pressure were assessed using a validated electronic sphygmomanometer (Omron M6 HEM‐7211‐E8(V)). Blood pressure percentiles were calculated according to sex, age and height‐specific reference data [34].

#### 2.5.3 Cardiorespiratory Endurance

Preteens completed the validated 20‐m shuttle run test to estimate cardiorespiratory endurance [35]. The last stage number completed was used as a proxy of cardiorespiratory endurance.

#### 2.5.4 Laboratory Analysis of Blood Biomarkers

For this analysis, we chose to include biomarkers that are routinely measured in large research studies and clinical settings. Non‐fasting serum concentrations of glucose, insulin, total cholesterol, high‐density lipoprotein cholesterol (HDL‐C), triglycerides and C‐reactive protein were analysed on the Cobas c701/702 module of the Roche Cobas 8000 analyser (Roche Diagnostics GmbH, Penzburg, Germany). Homeostatic Model Assessment for Insulin Resistance index and low‐density lipoprotein cholesterol were calculated using the appropriate standard formulas. Outliers of biomarkers more than 5 standard deviations from the mean were excluded.

#### 2.5.5 Foot Length Measurement

Foot length was measured to the nearest 0.1 cm using the Junior Brannock foot device (The Brannock Device Company, Liverpool, New York, the United States). Right and left foot measures were summed and averaged to provide a mean length. Preteens also completed a questionnaire that included a question phrased as ‘*When did you last get new trainers?’* and preteens were requested to recall the most applicable time period.

#### 2.5.6 Sexual Development

Mothers provided estimates of their preteens' sexual development using standardised Tanner staging figures [12]. Pubic hair distribution was used to estimate secondary sexual development in both sexes, and breast development was used to estimate primary sexual development in girls only. Testicular volume was not assessed in boys to assess primary sexual development. Preteens were dichotomised as being in Tanner Stage 1 or not (Stages 2–5).

### 2.6 Statistical Analysis

Statistical analysis was carried out using the IBM Statistical Package for the Social Sciences version 27.0 (Macintosh, Armonk, New York, the United States). Normality was assessed for all continuous variables using the Kolmogorov–Smirnov tests and visual inspection of simple histograms. Skewed data was log‐transformed and assessed again for normality before carrying out the appropriate parametric or non‐parametric test. Differences in characteristics between boys and girls and differences in foot length and shoe size between Tanner staging groups were explored using independent *t*‐tests for normally distributed variables, Mann–Whitney *U* tests for non‐normally distributed variables and *χ* ^2^ tests for categorical variables. Differences in responses to the acceptability questionnaire and data availability between boys and girls were explored using *χ* ^2^ tests only. Spearman's *ρ* correlations were used to examine unadjusted associations of blood pressure percentiles and cardiorespiratory endurance with blood biomarkers and of circumferences with body composition parameters.

Any result that was suggestive of an association was further investigated using linear regression models that were created using a forced entry approach with pairwise deletion, and the unstandardised Beta (*B*) coefficients were reported with a 95% confidence level. The a priori selection of covariates was informed by the literature [10, 12, 36], which included maternal ethnicity, socio‐economic status (Pobal Hasse and Pratshcke index score), child sex, breastfeeding exposure, preteen physical activity [37], sexual development, preteen BMI, and preteen height (selected covariates varied depending on the model of interest). Directed acyclic graphs were used to visualise assumptions regarding the selected covariates for the adjusted analyses (Figures S1–S3). Age of the preteen at the study visit and original trial group allocation were controlled for to account for potential differences in the population.

Missing data was handled using complete case analysis, and an outline of the percentage of missing data for each variable is provided in Table S1. The Benjamini–Hochberg correction method was applied to account for multiple testing with a false discovery rate of 0.05 due to the large number of statistical tests run for this analysis. Initially, *p* values of less than 0.05 were deemed significant for all analyses, before being converted to corresponding *q* values for the determination of true significance.

## 3 Results

### 3.1 Descriptive Characteristics From the ROLO Preteen Cohort

Cohort characteristics are displayed in Table 1. In total, 21.4% and 8.1% of preteens had overweight (BMI *z*‐score > 1.0) and obesity (BMI *z*‐score > 2.0), respectively. Based on maternal report, 87.5% of boys and 89.4% of girls were at Tanner Stage 1 of pubic hair distribution, and 78.7% of girls were at Tanner Stage 1 of breast development.

**Table 1:** Maternal and preteen characteristics from the ROLO longitudinal birth cohort.

	Total		Boys		Girls				
	*N*	Mean (SD)/Median (IQR)/*n* (%)	*N*	Mean (SD)/Median (IQR)/*n* (%)	*N*	Mean (SD)/Median (IQR)/*n* (%)	Effect Size/Estimate/OR (95% CI)	*p*	*q*
Maternal and infant characteristics									
Maternal ethnicity (White Irish), *n* (%)	408	375 (91.9)	204	190 (93.1)	204	185 (90.7)	1.39 (0.67, 2.86)	0.36	0.035
HP Index	408	7.5 (1.1, 12.5)	204	7.45 (0.3, 12.77)	204	7.55 (1.32, 11.8)	0.00 (−1.8, 1.6)	0.95^b^	0.048
RCT group (intervention), *n* (%)	408	205 (50.2)	204	104 (51.0)	204	101 (49.5)	1.06 (0.71, 1.56)	0.76	0.043
Child sex (male), *n* (%)	408	204 (50.0)	—	—	—	—	—	—	—
Breastfeeding exposure and duration									
Never, *n* (%)	362	134 (37.0)	179	74 (41.3)	183	60 (32.8)	—	0.002	0.020**
< 2 months, *n* (%)	362	46 (12.7)	179	19 (10.6)	183	27 (14.8)			
≥ 2 months and < 4 months, *n* (%)	362	39 (10.8)	179	28 (15.6)	183	11 (6.0)			
≥ 4 months, *n* (%)	362	143 (39.5)	179	58 (32.4)	183	85 (46.4)			
Preteen characteristics									
Age at follow‐up (years)	408	9.83 (9.24, 10.27)	204	9.87 (9.27, 10.28)	204	9.78 (9.22, 10.27)	0.07 (−0.03, 0.19)	0.17^b^	0.029
Pubic hair distribution									
Stage I, *n* (%)	321	284 (88.5)	160	140 (87.5)	161	144 (89.4)	0.82 (0.41, 1.64)	0.58	0.039
Breast development									
Stage I, *n* (%)	—	—	—	—	164	129 (78.7)	—	—	—
BMI *z*‐score	407	0.41 (1.08)	204	0.57 (1.1)	203	0.25 (1.03)	0.29 (0.1, 0.49)	0.003^a^	0.021**
Overweight, *n* (%)	407	87 (21.4)	204	49 (24.0)	203	38 (18.7)	—	0.018	0.023**
Obesity, *n* (%)	407	33 (8.1)	204	23 (11.3)	203	10 (4.9)			
Physical activity level (PAQ‐C)	372	2.48 (0.66)	183	2.59 (0.64)	189	2.37 (0.66)	0.32 (0.12, 0.52)	0.002^a^	0.021**

*Note:* Results presented as mean (SD) for normally distributed variables, median (IQR 25th, 75th percentile), or *n* (%) for categorical variables. *N* = total population with available data; *n* = frequency. *q* values correspond to the level of significance to which each *p* value is compared as part of the Benjamini–Hochberg adjustment.

Abbreviations: BMI, body mass index; HP, Haase and Pratschke; IQR, interquartile range; OR, odds ratio; PAQ‐C, Physical Activity Questionnaire for Older Children; RCT, randomised control trial; ROLO, Randomised cOntrol trial of a LOw glycaemic index diet in pregnancy to prevent macrosomia; SD, standard deviation.

** *q* < 0.023 is significant after adjustment for multiple testing.

^a^ *p* values determined using independent *t*‐tests for normally distributed variables.

^b^ *p* values determined using Mann–Whitney *U* test for non‐normally distributed variables. *χ* ^2^ tests were performed for categorical variables.

### 3.2 Feasibility and Acceptability of Research Methods at the ROLO Preteen Visit

Detailed description of complete data obtained at the ROLO 9–11‐year study visit is shown in Table S2. Of those who attended, a blood sample was not obtained for 47.8% of preteens, and Tanner staging assessment was incomplete for 21.3%. All other research methods obtained complete data for > 85% of participants. Reasons for missing data were not recorded. A breakdown of the approximate cost of the measurements and tests performed at the ROLO Preteen visit is provided in Table S3.

A subgroup of 50 preteens who attended a ROLO Preteen follow‐up appointment and obtained at least one measurement completed an acceptability questionnaire after their visit (Table 2). Description of complete data obtained for this subgroup is detailed in Table S2. No preteens rated the study measurements, exercises or online health questionnaires as ‘unacceptable’ or ‘very unacceptable’. Of 40 preteens, 5% (*n* = 2) rated providing a blood sample as ‘unacceptable’ or ‘very unacceptable’.

**Table 2:** Acceptability of research methods at 9–11 years of age.

	Acceptability questionnaire (*n* = 50)				
	Total	Boys	Girls	*p*	*q*
How acceptable were the measurements?					
Very acceptable, *n* (%)	32 (65.3)	11 (55.0)	21 (72.4)	0.08	0.026
Acceptable, *n* (%)	14 (28.6)	6 (30.0)	8 (27.6)		
Neutral, *n* (%)	3 (6.1)	3 (15.0)	0 (0)		
Unacceptable, *n* (%)	0 (0)	0 (0)	0 (0)		
Completely unacceptable, *n* (%)	0 (0)	0 (0)	0 (0)		
How acceptable were the exercises?					
Very acceptable, *n* (%)	27 (56.3)	13 (65.0)	14 (50.0)	0.58	0.040
Acceptable, *n* (%)	18 (37.5)	6 (30.0)	12 (42.9)		
Neutral, *n* (%)	3 (6.3)	1 (5.0)	2 (7.1)		
Unacceptable, *n* (%)	0 (0)	0 (0)	0 (0)		
Completely unacceptable, *n* (%)	0 (0)	0 (0)	0 (0)		
How acceptable was getting blood taken?					
Very acceptable, *n* (%)	10 (25.0)	5 (31.3)	5 (20.8)	0.26	0.031
Acceptable, *n* (%)	14 (35.0)	3 (18.8)	11 (45.8)		
Neutral, *n* (%)	14 (35.0)	7 (43.8)	7 (29.2)		
Unacceptable, *n* (%)	1 (2.5)	1 (6.3)	0 (0)		
Completely unacceptable, *n* (%)	1 (2.5)	0 (0)	1 (4.2)		
How acceptable were the questionnaires?					
Very acceptable, *n* (%)	25 (50.0)	10 (47.6)	15 (51.7)	0.49	0.037
Acceptable, *n* (%)	24 (48.0)	10 (47.6)	14 (48.3)		
Neutral, *n* (%)	1 (2.0)	1 (4.8)	0 (0)		
Unacceptable, *n* (%)	0 (0)	0 (0)	0 (0)		
Completely unacceptable, *n* (%)	0 (0)	0 (0)	0 (0)		

*Note:* Results presented as *n* (%) for categorical variables. *p* values determined using Pearson's *χ* ^2^ tests. *q* values correspond to the level of significance to which each *p* value is compared as part of the Benjamini–Hochberg adjustment. **q < 0.023 significant after adjustment for multiple testing.

### 3.3 Public and Patient Involvement With the ROLO YPAG

In total, 13 participants (age range 12–17 years) attended the fifth YPAG session. Of these, 8 attendees were study children (age range 12–16 years old) who had all participated in the ROLO Preteen study, and the remainder were older siblings (age range 13–17 years old) of the study children. Discussions were prompted with images of health measurements on a PowerPoint slide.

#### 3.3.1 Measures of Anthropometry

Firstly, researchers prompted the group to share their opinions about various measures of anthropometry. Study children were asked to reflect on their experience at their most recent study visit, and siblings were also encouraged to share their views. The majority (77%) agreed that neck circumference was an easy measurement of adiposity, although one participant viewed it as uncomfortable.

I'd say it's easier.

Older sibling 1, aged 17 years

I feel like I'd just be a bit uncomfortable if I had something around my neck.

Study child 1, aged 12 years

#### 3.3.2 Measures of Cardiovascular and Metabolic Health

Secondly, when prompted about their preferences for various measures of cardiovascular health, the consensus of the group (73%) was a preference for blood pressure due to its less invasive nature of assessment that does not require possibly painful venepuncture with a needle.

You don't need a needle going in.

Older sibling 2, aged 14 years

Despite this, the group felt that a blood sample provides more information on cardiovascular and metabolic health status that may not be obtained from less invasive procedures.

I think it's because you can tell more from it. I think you get more answers and stuff from it.

Study child 2, aged 16 years

#### 3.3.3 Measures of Cardiorespiratory Endurance

Finally, when the group were prompted for their views on measures of cardiorespiratory endurance, the YPAG described the 20‐m shuttle run test as an easy assessment method compared to more extensive and invasive methods such as the maximal oxygen consumption treadmill test.

It's easier to carry out.

Study child 3, aged 14 years

### 3.4 Feasibility of Blood Pressure and Cardiorespiratory Endurance as Metabolic Indicators in Preteens

Significant correlations between blood pressure percentiles and cardiorespiratory endurance with laboratory biomarkers in Table S4 were further explored in multiple linear regression models in Table 3. Initially, after controlling for covariates, there was a weak inverse association between diastolic blood pressure percentile and HDL‐C (mmol/L) in boys only (*B* = −0.004, 95% confidence interval [CI] = −0.01, 0.00, *p* = 0.035, *q* = 0.024) (Table 3). This association did not remain after adjustment for multiple testing.

**Table 3:** Adjusted associations between blood pressure percentiles, cardiorespiratory endurance and laboratory biomarkers.

	*B*	Adj. *R* ^2^	95% CI	*p*	*q*
Systolic blood pressure percentile					
Boys^a^					
HDL‐C (mmol/L)	−0.003	0.04	(−0.01, 0.001)	0.10	0.027
Diastolic blood pressure percentile					
Boys^a^					
HDL‐C (mmol/L)	−0.004	0.06	(−0.01, 0.00)	0.035	0.024
Cardiorespiratory endurance (20‐m shuttle run test score)					
Total group^b^					
TC (mmol/L)	−0.06	−0.08	(−0.19, 0.07)	0.34	0.034
LDL‐C (mmol/L)	−0.04	−0.06	(−0.15, 0.05)	0.37	0.035
Non‐HDL‐C (mmol/L)	−0.03	−0.05	(−0.14, 0.43)	0.32	0.033
CRP (mg/L)*	−0.01	0.11	(−0.10, 0.08)	0.84	0.045
Boys^a^					
LDL‐C (mmol/L)	−0.06	0.04	(−0.15, 0.03)	0.19	0.029
Non‐HDL‐C (mmol/L)	−0.05	0.04	(−0.16, 0.05)	0.31	0.032
CRP (mg/L)*	−0.01	0.13	(−0.08, 0.08)	0.98	0.049
Girls^a^					
CRP (mg/L)*	−0.01	0.13	(−0.12, 0.08)	0.73	0.043

Abbreviations: Adj., adjusted; CI, confidence interval; CRP, C‐reactive protein; LDL‐C, low‐density lipoprotein cholesterol; non‐HDL‐C, non‐high‐density lipoprotein cholesterol; TC, total cholesterol.

* Log10 transformed data was used.

^a^ Models adjusted for age at follow‐up, study group allocation, sexual development, breastfeeding, HP index, preteen BMI and preteen physical activity.

^b^ Models adjusted for child sex, age at follow‐up, study group allocation, sexual development, breastfeeding, HP index, preteen BMI and preteen physical activity. *q* values correspond to the level of significance to which each *p* value is compared as part of the Benjamini–Hochberg adjustment. **q < 0.023 significant after adjustment for multiple testing.

### 3.5 Feasibility of Foot Length to Predict Stage of Sexual Maturity in Preteens

Significant differences in Table S5 were explored further using multiple regression models after adjustment for potential covariates. Initially in adjusted analyses, boys in Tanner Stages 2–5 of pubic hair distribution had 1 cm longer feet than boys in Tanner Stage 1 of pubic hair distribution (95% CI = 0.12, 1.88, *p* = 0.026, *q* = 0.023) (Table 4). This association did not remain after adjustment for multiple testing.

**Table 4:** Adjusted associations between foot length and stages of sexual development.

	Tanner stages 2–5				
	*B*	Adj. *R* ^2^	95% CI	*p*	*q*
Girls: Breast development					
Average foot length (cm)	−0.43	0.70	(−1.18, 0.30)	0.24	0.030
Girls: Pubic hair distribution					
Average foot length (cm)	0.88	0.70	(−0.05, 1.82)	0.06	0.035
Boys: Pubic hair distribution					
Average foot length (cm)	1.00	0.74	(0.12, 1.88)	0.026	0.023

*Note:* Values determined using linear regression models. Tanner Stage 1 was used as a reference to which Tanner Stages 2–5 were compared. All models adjusted for age at study visit, study group allocation, maternal ethnicity and preteen height. *q* values correspond to the level of significance to which each *p* value is compared as part of the Benjamini–Hochberg adjustment. **q < 0.023 significant after adjustment for multiple testing.

Abbreviations: Adj, adjusted; CI, confidence interval.

### 3.6 Feasibility of Anthropometry for the Estimation of Body Composition in Preteens

Significant correlations in Table S6 were further explored in multiple regression models after adjustment for covariates. Neck and mid‐upper arm circumference were positively associated with all body composition measures in the total group (Table 5). Significant associations were found for all models when stratified by sex (data not shown). All associations remained significant after adjustment for multiple testing.

**Table 5:** Adjusted associations between neck and mid‐upper arm circumference with body composition parameters.

	Total group				
	*B*	Adj. *R* ^2^	95% CI	*p*	*q*
Neck circumference (cm)					
BMI *z*‐score	0.42	0.48	(0.34, 0.49)	< 0.001	0.015**
WC (cm)	2.83	0.48	(2.22, 3.45)	< 0.001	0.016**
SSF (mm)	4.84	0.55	(3.96, 5.72)	< 0.001	0.014**
Total fat mass (kg)	1.86	0.59	(1.52, 2.20)	< 0.001	0.015**
VAT (cm^2^)^a^	3.31	0.40	(2.45, 4.17)	< 0.001	0.018**
Body fat (%)	2.26	0.40	(1.66, 2.87)	< 0.001	0.018**
Mid‐upper arm circumference (cm)					
BMI *z*‐score	0.32	0.75	(0.29, 0.35)	< 0.001	0.005**
WC (cm)	2.10	0.63	(1.78, 2.41)	< 0.001	0.010**
SSF (mm)	3.39	0.68	(2.94, 3.84)	< 0.001	0.010**
Total fat mass (kg)	1.45	0.83	(1.32, 1.58)	< 0.001	0.004**
VAT (cm^2^)^a^	2.45	0.52	(1.99, 2.92)	< 0.001	0.015**
Body fat (%)	2.05	0.72	(1.80, 2.30)	< 0.001	0.008**

*Note:* Values determined using linear regression models. All models adjusted for child sex, study group allocation, age at follow‐up, sexual development, preteen physical activity, HP index, maternal ethnicity and breastfeeding exposure. *q* values correspond to the level of significance to which each *p* value is compared as part of the Benjamini–Hochberg adjustment.

Abbreviations: Adj, adjusted; BMI, body mass index; CI, confidence interval; SSF, sum of skinfolds; VAT, visceral adipose tissue; WC, waist circumference.

^a^ VAT estimated using the sex‐specific prediction equations proposed by Samouda et al.

* *q* < 0.023 significant after adjustment for multiple testing.

## 4 Discussion

### 4.1 Main Findings

Few studies have investigated youth's acceptability of health research methods, and our novel study attempts to fill this gap in an Irish context. Research methods that are disliked by participants are more likely to be left incomplete [38]. Greater participant satisfaction improves retention, quality of results, and attendance at follow‐up visits [5]. Overall, the research methods utilised in the ROLO Preteen study received a high rating of acceptability. Circumferences of the mid‐upper arm and neck were positively associated with body composition parameters.

### 4.2 Interpretation

In the ROLO Preteen study, analysis of the proportion of complete data indicated that the majority of research methods were acceptable to participants. This is consistent with our findings that no preteen rated the study measurements, exercises or questionnaires as unacceptable research methods for assessing health. This is important, given that all participants were informed by the researchers that they were free to opt out of any component of the study visit. In terms of feasibility, the most complete data was obtained for anthropometric measures, which are quick, easy to perform and only require a simple measuring tape or portable tools. While we did not record the reasons for missing data, it is likely that factors such as sitting still for longer periods, specialist training and reliance on equipment contributed to incomplete DXA scans and blood pressure measurements, along with the additional space required to complete the 20‐m shuttle run. Our findings are consistent with other studies that reported high rates of satisfaction among young people participating in health‐related research [5, 39], which has important implications for future research design and delivery.

A blood sample was obtained for half of the preteens who attended a study visit, and we observed undertones of blood work phobia in the ROLO cohort, with the use of ‘needle’ terminology in YPAG discussions. Recent qualitative research reported that blood sampling was the most burdensome test for all children participating in a clinical trial, whereas the discomfort of other procedures, such as urine sampling and ultrasound, varied [8]. It is also possible that some ROLO participants may not have had the opportunity to provide a blood sample if a phlebotomist was not available at the time of their visit. When we analysed whether blood pressure and cardiorespiratory endurance testing correlated with blood markers, we initially found that a higher diastolic blood pressure percentile was associated with lower HDL‐C in boys only. However, the association was no longer significant after adjusting for multiple testing. Despite its invasive nature, blood sampling plays an important role in clinical and research settings, and it is plausible that less invasive methods may not suffice as surrogate indicators of metabolic risk. Of those who provided a blood sample in our cohort, only 5% rated it as an unacceptable research method, and the youth also acknowledged the valuable information provided by a blood sample in the YPAG discussions. This is consistent with the opinions of parents from the ROLO Family Advisory Committee, who were positive about the use of a blood test in childhood to predict health outcomes [40]. Parents described it as a relevant and useful method to determine health status, compared to other tests.

Of those who attended a ROLO Preteen study visit, 20% did not complete the Tanner staging assessment. Previous qualitative research exploring children's self‐reported discomforts as participants in clinical research found that Tanner staging assessments can cause embarrassment [2]. The use of foot length may have an advantage over the Tanner stage as a novel and non‐invasive approach to estimate pubertal onset [13]. A lay panel of 10 young people aged 10–16 years agreed that the measurement of foot size was acceptable as a different approach to pubertal assessment [12]. Physical growth in the preteen period begins distally, and foot length is one of the earliest and most easily detectable changes before the onset of puberty, because it occurs before the pubertal height spurt [16]. Initially, in adjusted analyses, we found longer foot length remained associated with Tanner Stages 2–5 of pubic hair development in boys only; however, it did not remain significant after adjustment for multiple testing, and we were unable to replicate this association with testicular volume to strengthen the results. It is likely that the correlation between foot length and sexual maturity in girls was null because adrenarche typically occurs earlier, between the ages of 7–9 years. Another straightforward question that we explored in our analysis is for parents and preteens to recall a recent change in footwear, indicating growing foot size, rather than remembering when the appearance of breast development or pubic hair began [17].

While we acknowledge the global importance of BMI as a practical and cost‐effective tool for obesity screening [20], our findings attempt to highlight the supplementary usefulness of alternative methods that are inexpensive, easy to use and acceptable to preteens. Circumferences of the neck and mid‐upper arm have no variation throughout the day, do not require a calibrated scale, and are less associated with weight or the abdominal area [41]. This is based on previous research that suggests these measures may be more associated with embarrassment in this age group [18]. In another qualitative study, adolescents highlighted that routine weight assessment by a doctor may be embarrassing or cause them to feel bad, particularly among those living with overweight or obesity, and some preferred that this topic be addressed using alternative measures of adiposity [42]. Detailed body composition analysis techniques are operationally costly and require specialist training, which limits their feasibility. We found circumferences of the neck and mid‐upper arm were strongly associated with multiple parameters of adiposity at 9–11 years of age after controlling for potential covariates, in line with previous research [21, 22].

### 4.3 Strengths and Limitations

The strengths of this exploratory study include a moderate sample size of 9–11‐year‐olds with equal gender distribution and detailed information on several aspects of preteen health, including body composition, cardiometabolic profile and sexual development. A combination of accurate objective anthropometry and body composition measurements was collected by trained researchers. Complete data was available for a large proportion of the cohort. The inclusion of laboratory metabolic and inflammatory parameters enabled greater insight into their cardiometabolic profiles at this time point in development. Including the views of youth who took part in public and patient involvement with the ROLO YPAG strengthens the acceptability and feasibility of research methods used in the ROLO Preteen study.

This study has several limitations. The acceptability questionnaire may have been influenced by acquiescent response bias, which we were unable to account for [43]. In addition, a generic acceptability questionnaire was used, which limited our ability to determine their acceptability of individual measurements. Although most preteens who completed the acceptability questionnaire had complete data, responses from participants who did not complete all aspects of the study may be subject to inherent bias. Our findings are limited to a generally healthy preteen cohort; thus, they may not be applicable to other populations or age groups. Given the poor fit of some of the models, which is likely due in part to the small sample size, these results should be interpreted with caution. We did not record information on reasons for missing data, which would have strengthened the argument of feasibility. This analysis is limited to one measurement of foot length at a single time point, and we acknowledge that serial measures may provide a more accurate indication of sudden growth changes related to pubertal onset [16].

### 4.4 Implications and Future Research

The findings from this study may inform best practice or ethical guidelines for conducting health assessments with young people, which prioritise enjoyable and feasible health‐related experiences for youth. Flexible health assessments that allow options and choice for young people that best suit their needs could be incorporated into child health screening and surveillance services in school programmes and the community [44]. Clinicians and researchers may benefit from exploring minimally invasive approaches for youth who are reluctant or fearful of some procedures, such as blood sampling. Previous work has highlighted the importance of strategies to ease blood work practices, including topical anaesthetic cream, micro‐sampling [45], distraction techniques [46] or augmented reality tools [47], which are supported by youth in the literature.

The findings of this analysis highlight the importance of further investigating the feasibility of quick and simple acceptability or feedback questionnaires for youth in clinical settings, such as after routine health check‐ups or a medical procedure. This opportunity may allow for improvements to be made to healthcare experiences. This study explored acceptability at a single follow‐up visit, but further studies could build on this study and capture qualitative and quantitative acceptability data across multiple study visits in childhood and adolescence to learn whether views change as young participants age. Future research may also benefit from asking youth about the acceptability of health research methods earlier in the research process, so that feasible and less burdensome procedures could be incorporated from the onset of the study.

## 5 Conclusion

This exploratory study provides new insights into research method acceptability to preteens participating in a longitudinal birth cohort study. Health research methods were acceptable to preteens. Circumferences of the mid‐upper arm and neck were positively associated with body composition parameters. Our novel findings may help researchers and clinicians when designing acceptable and feasible health examinations for youth. Future exploration of minimally invasive measures of health with low burden may increase youth participation in medical research and translate to more enjoyable healthcare experiences.

## Author Contributions

**Sophie Callanan:** writing – original draft, conceptualization, investigation, methodology, formal analysis, and data curation. **Anna Delahunt:** writing – review and editing, methodology. **Alex O. Start:** writing – review and editing, resources. **Rachel K. Crowley, Patrick J. Twomey, Ciara M. McDonnell, Catherine M. Phillips and Declan Cody:** writing – review and editing. **Fionnuala M. McAuliffe:** supervision, writing – review and editing, investigation, funding acquisition, methodology, and conceptualization. All authors approved the final version of the manuscript and agree to be accountable for all aspects of the work.

## Disclosure

The funders had no role in study design, data collection and analysis, decision to publish, or preparation of the manuscript.

## Ethics Statement

Ethical approval for the primary ROLO trial was obtained from the NMH Ethics Committee, Dublin, Ireland (GEN/279/12). Ethical approval for the 9‐11 year follow‐up was obtained from University College Dublin, Office of Research Ethics Committee, Dublin, Ireland (LS‐15‐06‐Geraghty‐McAuliffe).

## Consent

Informed, written maternal consent was obtained before study participation, and verbal assent was obtained from the study child.

## Conflicts of Interest

The authors declare no conflicts of interest.

## Supporting information


**Supplementary Figure 1:** Directed acyclic graphs of blood pressure percentiles and cardiorespiratory endurance with cardiometabolic biomarkers. **Supplementary Figure 2:** Directed acyclic graph of sexual development and foot length. **Supplementary Figure 3:** Directed acyclic graph of anthropometric circumferences and body composition parameters. **Supplementary Table 1:** Outline of missing data. **Supplementary Table 2:** Availability of data collected at the ROLO Preteen follow‐up. **Supplementary Table 3:** Cost of measurement equipment and testing for the ROLO Preteen study visits. **Supplementary Table 4:** Correlations between blood pressure percentiles, cardiorespiratory endurance, and laboratory biomarkers. **Supplementary Table 5:** Differences in foot size between stages of sexual development. **Supplementary Table 6:** Correlations between neck and mid‐upper arm circumference with body composition parameters.

## Data Availability

The data that support the findings of this study are available from the corresponding author upon reasonable request.
