# Supplementary material for: Acceptability and Feasibility of Health Measures in Preteens: Findings From the ROLO Longitudinal Birth Cohort Study
Source: Health Expect. 2025 Jul 28;28(4):e70359. doi: 10.1111/hex.70359 (PMC12301627; doi:10.1111/hex.70359)
Supplement: Supplementary file 1 — Supplementary Figure 1: Directed acyclic graphs of blood pressure percentiles and cardiorespiratory endurance with cardiometabolic biomarkers. Supplementary Figure 2: Directed acyclic graph of sexual development and foot length. Supplementary Figure 3: Directed acyclic graph of anthropometric circumferences and body composition parameters. Supplementary Table 1: Outline of missing data. Supplementary Table 2: Availability of data collected at the ROLO Preteen follow‐up. Supplementary Table 3: Cost of measurement equipment and testing for the ROLO Preteen study visits. Supplementary Table 4: Correlations between blood pressure percentiles, cardiorespiratory endurance, and laboratory biomarkers. Supplementary Table 5: Differences in foot size between stages of sexual development. Supplementary Table 6: Correlations between neck and mid‐upper arm circumference with body composition parameters. [file HEX-28-e70359-s001.docx]

**Acceptability and feasibility of health measures in preteens: findings from the ROLO longitudinal birth cohort study**

**Supplementary Material**

**A**
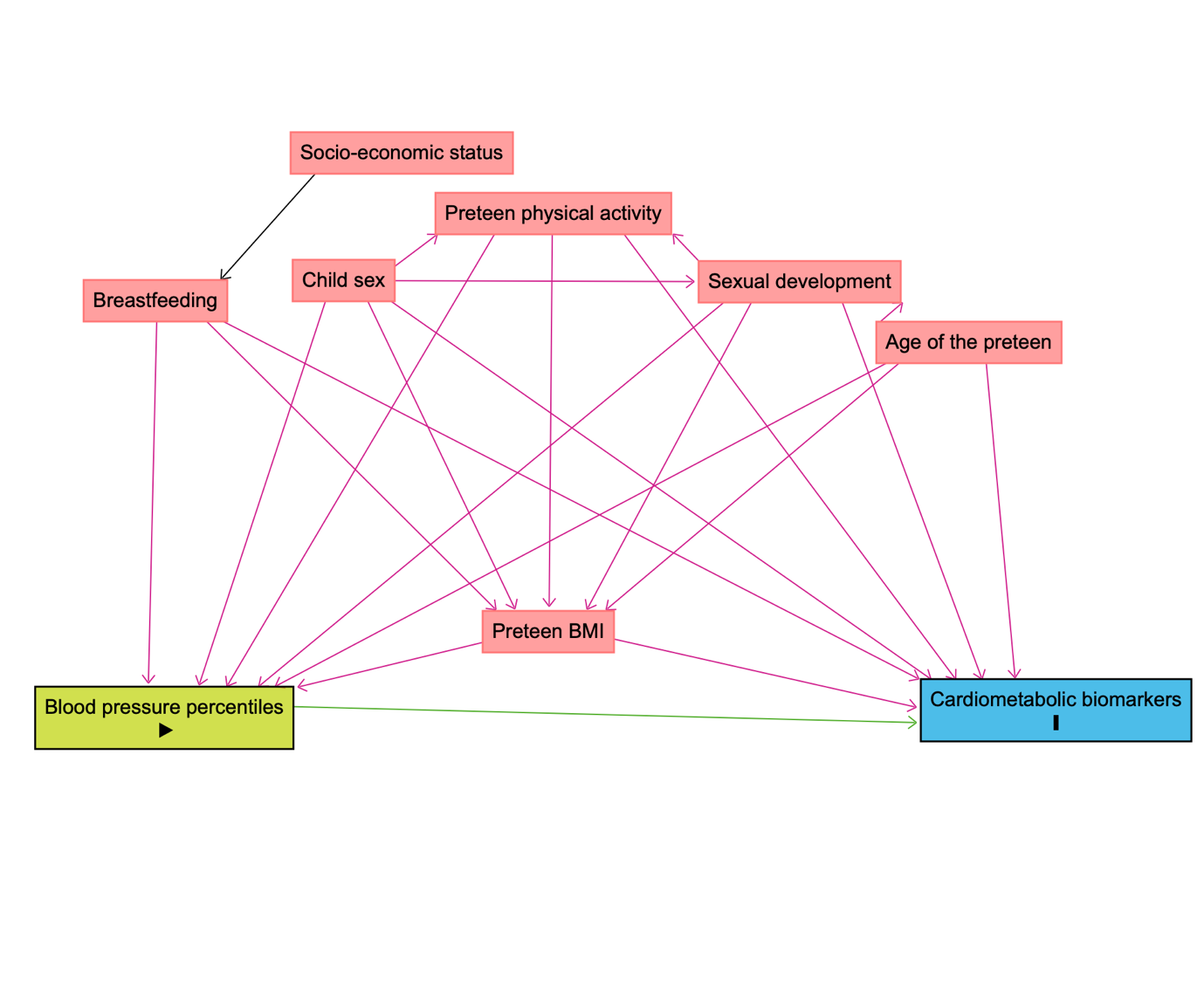


**B**
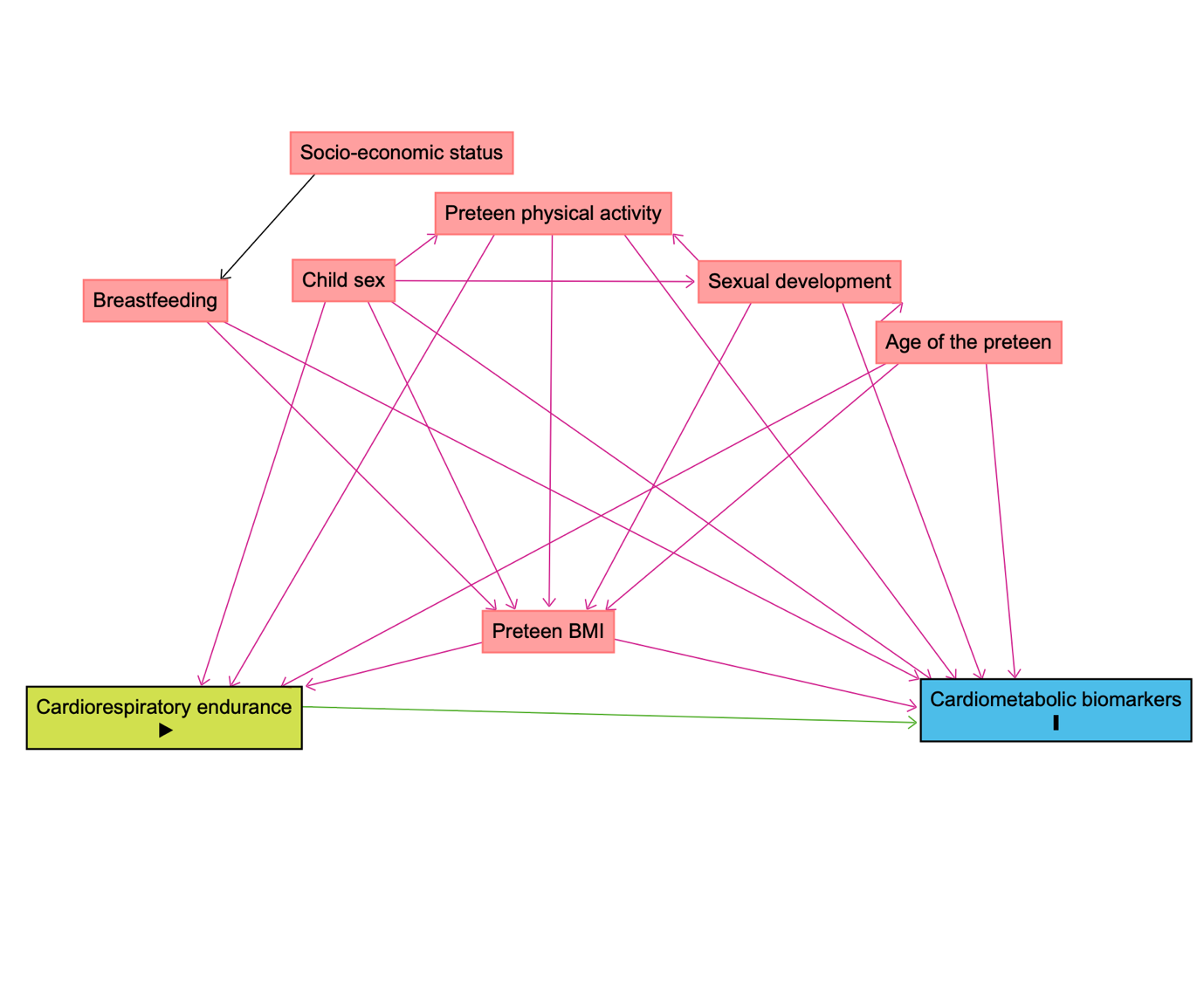


**Supplementary Figure 1.** Directed acyclic graphs of blood pressure percentiles and cardiorespiratory endurance with cardiometabolic biomarkers.


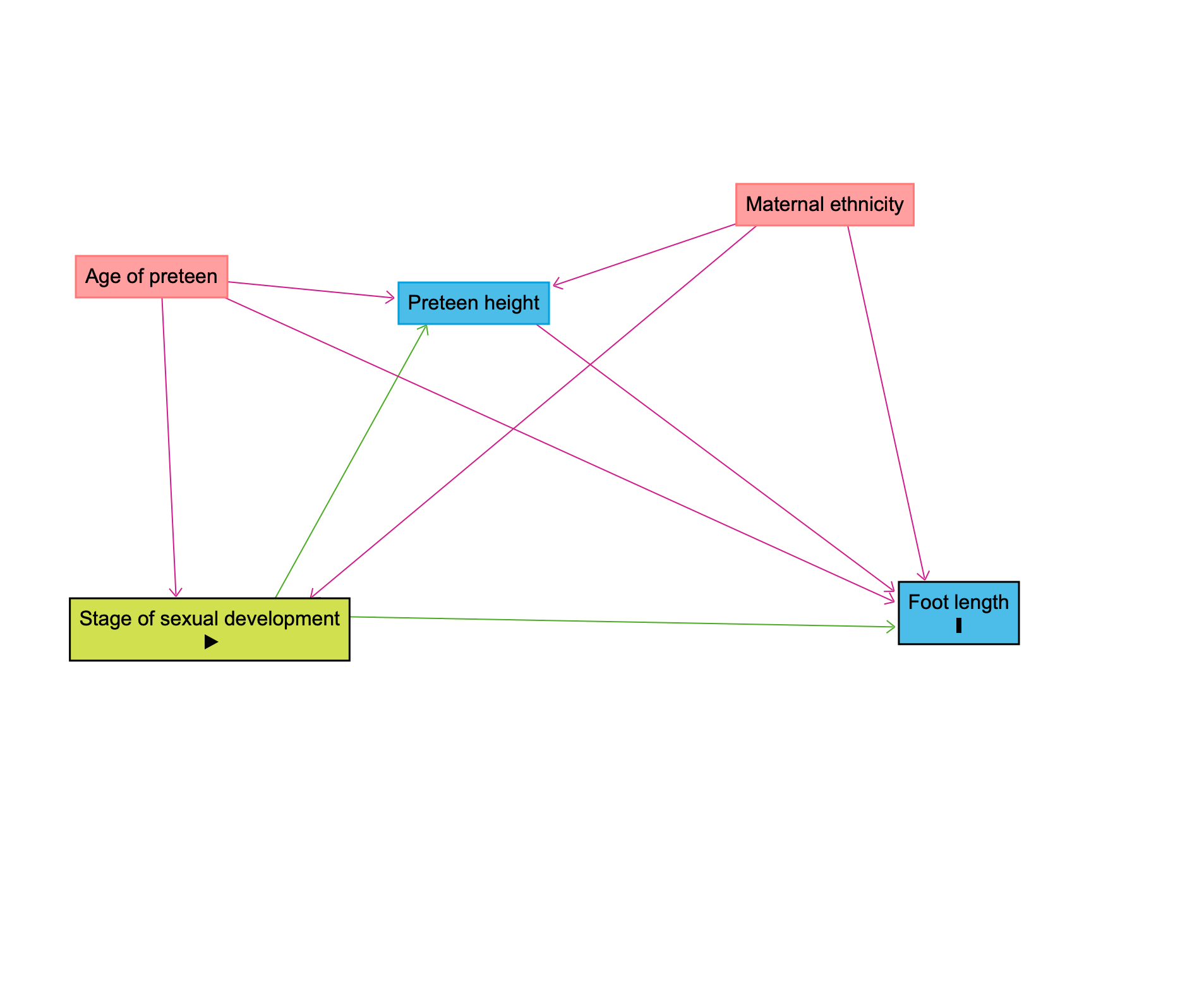


**Supplementary Figure 2.** Directed acyclic graph of sexual development and foot length.

**
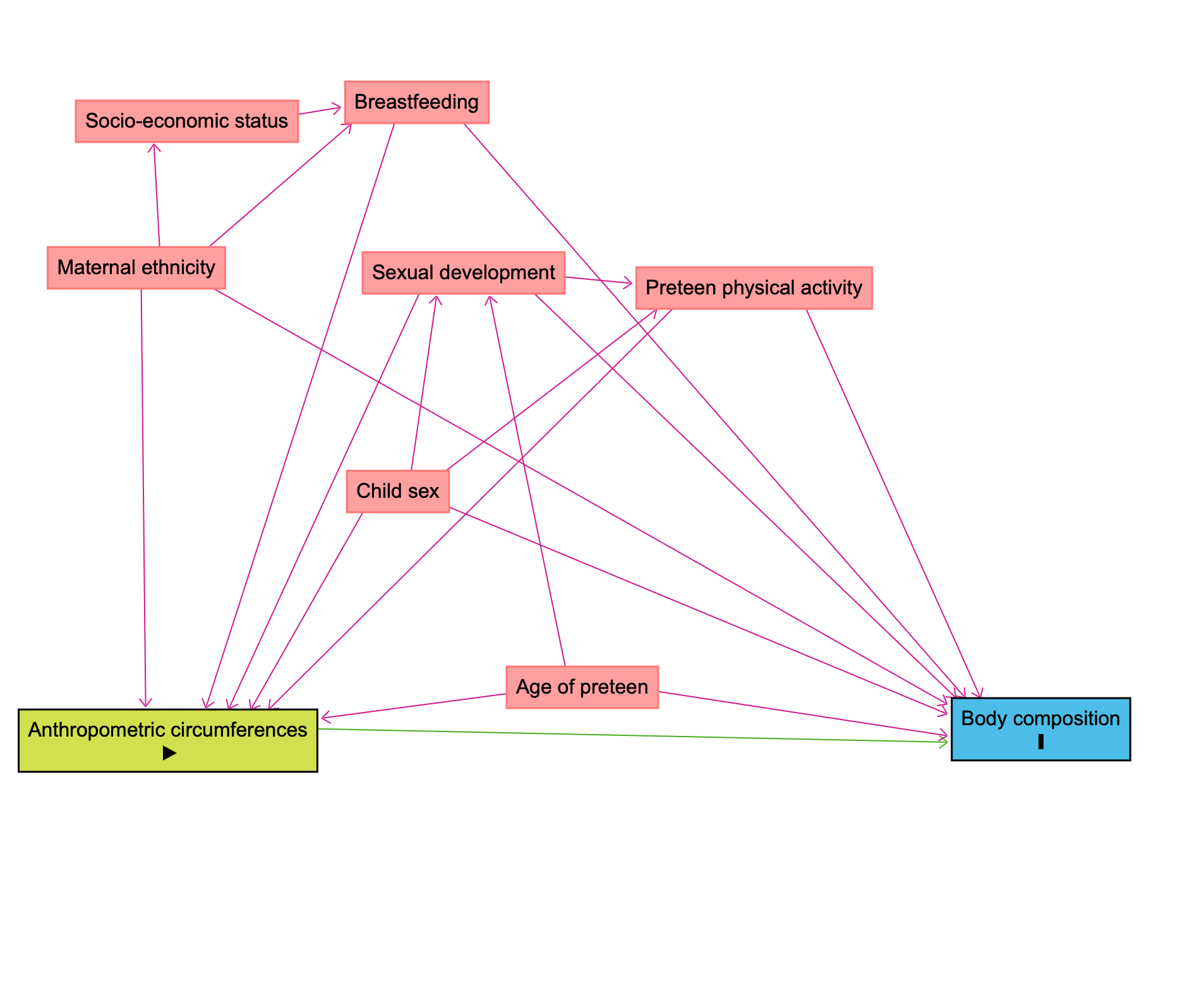
**

**Supplementary Figure 3.** Directed acyclic graph of anthropometric circumferences and body composition parameters.

| Supplementary Table 1. Outline of missing data. | | |
| --- | --- | --- |
| Variable | **Cases with missing data** | **Percentage of missing data** |
| Maternal ethnicity | 0 | 0.0% |
| Pobal Haase and Pratschke Index | 0 | 0.0% |
| Randomised control trial group | 0 | 0.0% |
| Child sex | 0 | 0.0% |
| Breastfeeding exposure | 46 | 11.2% |
| Age at follow-up | 0 | 0.0% |
| Pubic hair distribution | 87 | 21.3% |
| Breast development | 244 | 59.8% |
| Body mass index z-score | 1 | 0.2% |
| Physical activity level (PAQ-C score) | 36 | 8.8% |
| Acceptability questionnaire | 358 | 87.7% |
| Height | 0 | 0.0% |
| Weight | 1 | 0.2% |
| Waist circumference | 0 | 0.0% |
| Neck circumference | 0 | 0.0% |
| Mid-upper arm circumference | 4 | 1.0% |
| Sum of skinfolds | 40 | 9.8% |
| Visceral adipose tissue | 1 | 0.2% |
| Total fat mass | 58 | 14.2% |
| Body fat % | 58 | 14.2% |
| Average foot length | 61 | 15.0% |
| Shoe size questionnaire | 96 | 23.5% |
| Systolic blood pressure percentile | 26 | 6.4% |
| Diastolic blood pressure percentile | 26 | 6.4% |
| 20-M shuttle run test score | 28 | 6.9% |
| Total cholesterol | 196 | 48.0% |
| Low density lipoprotein cholesterol | 196 | 48.0% |
| High density lipoprotein cholesterol | 195 | 47.8% |
| Non-high density lipoprotein cholesterol | 196 | 48.0% |
| Triglycerides | 195 | 47.8% |
| Homeostatic Model of Assessment for Insulin Resistance | 198 | 48.5% |
| C-reactive protein | 206 | 50.5% |
| Abbreviations: PAQ-C Physical activity Questionnaire for Older Children. | | |

| Supplementary Table 2. Availability of data collected at the ROLO Preteen follow-up. | | | | | | |
| --- | --- | --- | --- | --- | --- | --- |
|  | **Total** | **Boys** | **Girls** | **OR (95% CI)** | ***p*** | ***q*** |
| Availability of data collected for the total cohort (N = 408) | | | | | | |
| Height, n (%) | 408 (100) | 204 (100) | 204 (100) | - | - | - |
| Weight, n (%) | 407 (99.8) | 204 (100) | 203 (99.5) | 0.49 (0.45, 0.55) | 0.31 | 0.032 |
| Waist circumference, n (%) | 408 (100) | 204 (100) | 204 (100) | - | - | - |
| Neck circumference, n (%) | 408 (100) | 204 (100) | 204 (100) | - | - | - |
| MUAC, n (%) | 404 (99.0) | 200 (98.0) | 204 (100) | 0.49 (0.44, 0.54) | 0.044 | 0.025 |
| Skinfolds, n (%) | 380 (93.1) | 189 (92.6) | 191 (93.6) | 0.85 (0.39, 1.85) | 0.69 | 0.041 |
| DXA, n (%) | 350 (85.7) | 178 (87.3) | 172 (84.3) | 1.27 (0.72, 2.22) | 0.39 | 0.035 |
| Foot length, n (%) | 347 (85.0) | 172 (84.3) | 175 (85.8) | 0.89 (0.51, 1.53) | 0.67 | 0.041 |
| Blood pressure, n (%) | 382 (93.6) | 193 (94.6) | 189 (92.6) | 1.39 (0.62, 3.11) | 0.41 | 0.036 |
| 20-M SRT, n (%) | 380 (93.1) | 189 (92.6) | 191 (93.6) | 0.85 (0.39, 1.85) | 0.69 | 0.042 |
| Blood sample, n (%) | 213 (52.2) | 115 (56.4) | 98 (48.0) | 1.39 (0.94, 2.06) | 0.09 | 0.027 |
| Health questionnaires, n (%) | 372 (91.1) | 183 (89.7) | 189 (92.6) | 0.69 (0.34, 1.38) | 0.29 | 0.031 |
| Tanner staging, n (%) | 321 (78.7) | 160 (78.4) | 161 (78.9) | 0.97 (0.6, 1.56) | 0.90 | 0.046 |
| Availability of data collected for the subgroup (N = 50) that completed the acceptability questionnaire | | | | | | |
| Height, n (%) | 50 (100) | 21 (100) | 29 (100) | - | - | - |
| Weight, n (%) | 50 (100) | 21 (100) | 29 (100) | - | - | - |
| Waist circumference, n (%) | 50 (100) | 21 (100) | 29 (100) | - | - | - |
| Neck circumference, n (%) | 50 (100) | 21 (100) | 29 (100) | - | - | - |
| MUAC, n (%) | 50 (100) | 21 (100) | 29 (100) | - | - | - |
| Skinfolds, n (%) | 48 (96.0) | 20 (95.2) | 28 (96.6) | 0.71 (0.04, 12.1) | 0.81 | 0.045 |
| DXA, n (%) | 49 (98.0) | 20 (95.2) | 29 (100) | 0.4 (0.29, 0.57) | 0.23 | 0.029 |
| Foot length, n (%) | 50 (100) | 21 (100) | 29 (100) | - | - | - |
| Blood pressure, n (%) | 50 (100) | 21 (100) | 29 (100) | - | - | - |
| 20-M SRT, n (%) | 50 (100) | 21 (100) | 29 (100) | - | - | - |
| Blood sample, n (%) | 31 (62.0) | 13 (61.9) | 18 (62.1) | 0.99 (0.31, 3.15) | 0.99 | 0.05 |
| Health questionnaires, n (%)^a^ | 41 (82.0) | 17 (81.0) | 24 (82.8) | 0.88 (0.2, 3.79) | 0.87 | 0.046 |
| Tanner staging, n (%)^b^ | 36 (72.0) | 18 (85.7) | 18 (62.1) | 3.66 (0.87, 15.3) | 0.06 | 0.026 |
| Abbreviations: ROLO Randomised cOntrol trial of a LOw glycaemic index diet in pregnancy to prevent macrosomia; OR Odds Ratio; MUAC Mid-upper arm circumference; DXA Dual-energy X-ray absorptiometry; 20-M SRT 20-metre shuttle run test. ^a^Completed by the preteen. ^b^Completed by the mother on behalf of the preteen. *q* values correspond to the level of significance to which each *p* value is compared to as part of the Benjamini-Hochberg adjustment, ***q*<0.023 significant after adjustment for multiple testing. | | | | | | |

| Supplementary Table 3. Cost of measurement equipment and testing for the ROLO Preteen study visits. | |
| --- | --- |
| Test / measurement equipment | **Cost** |
| Height Stadiometer (SECA 213) | €93.30* |
| Portable flat weighing scales (SECA 876) | €233.27* |
| Circumference tape (SECA 201) | €8.46* |
| Skinfold calipers (Holtain / Tanner) | €416.41* |
| Dual-energy x-ray absorptiometry scan (per scan) | €50.20 |
| Junior Brannock foot device (The Brannock Company) | €76.72 |
| Electronic sphygmomanometer | €58.46* |
| Bleep Lite Test smartphone application for 20-metre shuttle run test | Free |
| Phlebotomy fee (per hour) | €16.00 – €55.00 |
| Blood panel (laboratory analysis per person)** | €40.00 |
| *Excluding VAT.  **Includes analysis of lipid panel, glucose, insulin, and C-reactive protein. | |

| Supplementary Table 4. Correlations between blood pressure percentiles, cardiorespiratory endurance, and laboratory biomarkers. | | | | | | | | | | | | |
| --- | --- | --- | --- | --- | --- | --- | --- | --- | --- | --- | --- | --- |
|  | **Total** | | | | **Boys** | | | | **Girls** | | | |
|  | **n** | ***r*** | ***p*** | ***q*** | **n** | ***r*** | ***p*** | ***q*** | **n** | ***r*** | ***p*** | ***q*** |
| Systolic blood pressure percentile | | | | | | | | | | | | |
| TC (mmol/L) | 203 | -0.061 | 0.39 | 0.035 | 110 | -0.088 | 0.35 | 0.034 | 93 | -0.030 | 0.77 | 0.044 |
| LDL-C (mmol/L) | 203 | -0.075 | 0.28 | 0.031 | 110 | -0.055 | 0.56 | 0.039 | 93 | -0.080 | 0.44 | 0.036 |
| HDL-C (mmol/L) | 204 | -0.136 | 0.05 | 0.025 | 111 | -0.215 | 0.023 | 0.023** | 93 | -0.049 | 0.64 | 0.040 |
| Non-HDL-C (mmol/L) | 203 | 0.003 | 0.96 | 0.048 | 110 | 0.021 | 0.82 | 0.045 | 93 | 0.005 | 0.95 | 0.048 |
| TG (mmol/L) | 204 | 0.097 | 0.16 | 0.028 | 111 | 0.109 | 0.25 | 0.030 | 93 | 0.100 | 0.34 | 0.034 |
| HOMA-IR | 201 | 0.049 | 0.49 | 0.037 | 109 | 0.152 | 0.11 | 0.027 | 92 | -0.105 | 0.31 | 0.032 |
| CRP (mg/L) | 193 | -0.005 | 0.94 | 0.047 | 106 | 0.010 | 0.92 | 0.047 | 92 | 0.002 | 0.98 | 0.049 |
| Diastolic blood pressure percentile | | | | | | | | | | | | |
| TC (mmol/L) | 203 | -0.005 | 0.94 | 0.047 | 110 | -0.099 | 0.30 | 0.032 | 93 | 0.054 | 0.60 | 0.040 |
| LDL-C (mmol/L) | 203 | 0.040 | 0.57 | 0.039 | 110 | 0.020 | 0.83 | 0.045 | 93 | 0.028 | 0.79 | 0.044 |
| HDL-C (mmol/L) | 204 | -0.125 | 0.07 | 0.026 | 111 | -0.240 | 0.011 | 0.022** | 93 | 0.002 | 0.98 | 0.049 |
| Non-HDL-C (mmol/L) | 203 | 0.068 | 0.33 | 0.033 | 110 | 0.032 | 0.74 | 0.043 | 93 | 0.070 | 0.50 | 0.038 |
| TG (mmol/L) | 204 | 0.053 | 0.45 | 0.037 | 111 | 0.026 | 0.78 | 0.044 | 93 | 0.072 | 0.49 | 0.038 |
| HOMA-IR | 201 | 0.043 | 0.54 | 0.038 | 109 | 0.070 | 0.46 | 0.037 | 92 | 0.035 | 0.74 | 0.043 |
| CRP (mg/L) | 193 | 0.026 | 0.72 | 0.042 | 106 | -0.044 | 0.65 | 0.041 | 87 | 0.062 | 0.56 | 0.039 |
| Cardiorespiratory endurance (20-metre shuttle run test score) | | | | | | | | | | | | |
| TC (mmol/L) | 205 | -0.152 | 0.029 | 0.024 | 111 | -0.175 | 0.06 | 0.026 | 94 | -0.102 | 0.33 | 0.034 |
| LDL-C (mmol/L) | 205 | -0.144 | 0.040 | 0.025 | 111 | -0.228 | 0.016 | 0.023** | 94 | -0.007 | 0.94 | 0.048 |
| HDL-C (mmol/L) | 206 | 0.031 | 0.65 | 0.040 | 112 | 0.133 | 0.16 | 0.029 | 94 | -0.120 | 0.25 | 0.030 |
| Non-HDL-C (mmol/L) | 205 | -0.150 | 0.031 | 0.024 | 111 | -0.231 | 0.015 | 0.022** | 94 | -0.015 | 0.88 | 0.046 |
| TG (mmol/L) | 206 | -0.106 | 0.12 | 0.028 | 112 | -0.147 | 0.12 | 0.028 | 94 | -0.037 | 0.72 | 0.042 |
| HOMA-IR | 203 | -0.068 | 0.33 | 0.033 | 110 | -0.142 | 0.13 | 0.028 | 93 | 0.037 | 0.72 | 0.042 |
| CRP (mg/L) | 195 | -0.300 | <0.001 | 0.020** | 107 | -0.285 | 0.003 | 0.021** | 88 | -0.327 | 0.002 | 0.021** |
| Abbreviations: TC Total cholesterol; LDL-C Low-density lipoprotein cholesterol; HDL-C High-density lipoprotein cholesterol; TG Triglycerides; HOMA-IR Homeostatic Model of Assessment for Insulin Resistance; CRP C-reactive protein; 25-OHD 25-hydroxyvitamin D. All values determined using Spearman’s Rho correlations for non-normally distributed variables. *q* values correspond to the level of significance to which each *p* value is compared to as part of the Benjamini-Hochberg adjustment, ***q*<0.023 significant after adjustment for multiple testing. | | | | | | | | | | | | |

| Supplementary Table 5. Differences in foot size between stages of sexual development. | | | | | | | |
| --- | --- | --- | --- | --- | --- | --- | --- |
|  | **Tanner Stage 2 – 5** | | **Tanner Stage 1** | |  |  |  |
|  | **N** | **Mean (SD) /**  **n (%)** | **N** | **Mean (SD) /**  **n (%)** | **Effect size / Estimate**  **(95% CI)** | ***p*** | ***q*** |
| Girls: Breast Development | | | | | | | |
| Average foot length (cm) | 32 | 16.62 (2.83) | 122 | 14.38 (2.97) | -0.75 (-1.15, -0.35) | <0.001^a^ | 0.020** |
| *When did you last get new trainers?* | | |  |  |  |  |  |
| In the last 6 months, n (%) | 34 | 18 (52.9) | 111 | 70 (63.1) | - | 0.44 | 0.036 |
| Between 6 months – 1 year, n (%) | 34 | 9 (26.5) | 111 | 19 (17.1) |  |  |  |
| >1 year, n (%) | 34 | 7 (20.6) | 111 | 22 (19.8) |  |  |  |
| Girls: Pubic Hair Distribution | | | | | | | |
| Average foot length (cm)* | 15 | 17.5 (16.0, 19.0) | 136 | 14.25 (13.0, 16.5) | -0.92 (-1.47, -0.38) | <0.001^b^ | 0.020** |
| *When did you last get new trainers?* | |  |  |  |  |  |  |
| In the last 6 months, n (%) | 16 | 9 (56.3) | 126 | 77 (61.1) | - | 0.88 | 0.046 |
| Between 6 months – 1 year, n (%) | 16 | 3 (18.8) | 126 | 24 (19.0) |  |  |  |
| >1 year, n (%) | 16 | 4 (25.0) | 126 | 25 (19.8) |  |  |  |
| Boys: Pubic Hair Distribution | | | |  |  |  |  |
| Average foot length (cm) | 19 | 18.97 (3.96) | 131 | 16.49 (3.25) | -0.74 (-1.22, -0.25) | 0.003^a^ | 0.022** |
| *When did you last get new trainers?* | |  |  |  |  |  |  |
| In the last 6 months, n (%) | 17 | 15 (88.2) | 119 | 86 (72.3) | - | 0.25 | 0.031 |
| Between 6 months – 1 year, n (%) | 17 | 2 (11.8) | 119 | 18 (15.1) |  |  |  |
| >1 year, n (%) | 17 | 0 (0.0) | 119 | 15 (12.6) |  |  |  |
| Statistical comparisons determined using ^a^independent *t*-tests for normally distributed variables, ^b^Mann-Whitney U tests for non-normally distributed variables or Chi-square tests for categorical variables; *P*<0.05 considered statistically significant. N = available data; n = frequency. *q* values correspond to the level of significance to which each *p* value is compared to as part of the Benjamini-Hochberg adjustment, ***q*<0.023 significant after adjustment for multiple testing. | | | | | | | |

| Supplementary Table 6. Correlations between neck and mid-upper arm circumference with body composition parameters. | | | | | | | | | | | | |
| --- | --- | --- | --- | --- | --- | --- | --- | --- | --- | --- | --- | --- |
|  | **Total** | | | | **Boys** | | | | **Girls** | | | |
|  | **n** | ***r*** | ***p*** | ***q*** | **n** | ***r*** | ***p*** | ***q*** | **n** | ***r*** | ***p*** | ***q*** |
| Neck circumference (cm) | | | | | | | | | | | | |
| BMI z-score | 407 | 0.667 | <0.001 | 0.003** | 204 | 0.707 | <0.001 | 0.009** | 203 | 0.638 | <0.001 | 0.011** |
| WC (cm) | 408 | 0.669 | <0.001 | 0.003** | 204 | 0.718 | <0.001 | 0.008** | 204 | 0.628 | <0.001 | 0.011** |
| SSF (mm) | 368 | 0.554 | <0.001 | 0.009** | 185 | 0.733 | <0.001 | 0.009** | 183 | 0.639 | <0.001 | 0.012** |
| Total fat mass (kg) | 350 | 0.574 | <0.001 | 0.009** | 178 | 0.726 | <0.001 | 0.010** | 172 | 0.644 | <0.001 | 0.013** |
| VAT (cm^2^)^a^ | 407 | 0.448 | <0.001 | 0.013** | 204 | 0.718 | <0.001 | 0.008** | 203 | 0.507 | <0.001 | 0.017** |
| Body fat (%) | 350 | 0.398 | <0.001 | 0.017** | 178 | 0.630 | <0.001 | 0.014** | 172 | 0.479 | <0.001 | 0.018** |
| Mid-upper arm circumference (cm) | | | | | | | | | | | | |
| BMI z-score | 403 | 0.852 | <0.001 | <0.001** | 200 | 0.876 | <0.001 | 0.002** | 203 | 0.843 | <0.001 | 0.002** |
| WC (cm) | 404 | 0.783 | <0.001 | <0.001** | 200 | 0.830 | <0.001 | 0.003** | 204 | 0.752 | <0.001 | 0.006** |
| SSF (mm) | 366 | 0.778 | <0.001 | 0.001** | 183 | 0.815 | <0.001 | 0.004** | 183 | 0.750 | <0.001 | 0.007** |
| Total fat mass (kg) | 349 | 0.897 | <0.001 | <0.001** | 177 | 0.905 | <0.001 | 0.001** | 172 | 0.907 | <0.001 | 0.001** |
| VAT (cm^2^)^a^ | 403 | 0.705 | <0.001 | 0.002** | 200 | 0.830 | <0.001 | 0.003** | 203 | 0.595 | <0.001 | 0.014** |
| Body fat (%) | 349 | 0.795 | <0.001 | 0.002** | 177 | 0.829 | <0.001 | 0.004** | 172 | 0.813 | <0.001 | 0.006** |
| Abbreviations: BMI Body mass index; WC Waist circumference; SSF Sum of skinfolds; VAT Visceral adipose tissue. All values determined using Spearman’s Rho correlations for non-normally distributed variables. ^a^Estimated using the sex-specific VAT prediction equation proposed by Samouda *et al. q* values correspond to the level of significance to which each *p* value is compared to as part of the Benjamini-Hochberg adjustment, ***q*<0.023 significant after adjustment for multiple testing. | | | | | | | | | | | | |
